# Structural Alterations in Large-scale Brain Networks and Their Relationship with Sleep Disturbances in the Adolescent Population

**DOI:** 10.1038/s41598-020-60692-1

**Published:** 2020-03-02

**Authors:** Dajung Sung, Bumhee Park, Shin-Young Kim, Bung-Nyun Kim, Subin Park, Kyu-In Jung, Jungjin Kim, Min-Hyeon Park

**Affiliations:** 1 Department of Psychiatry, Eunpyeong St. Mary’s Hospital, College of Medicine, The Catholic University of Korea, Seoul, Republic of Korea; 2 Department of Biomedical Informatics, Ajou University School of Medicine, Suwon, Republic of Korea; 3 Office of Biostatistics, Ajou Research Institute for Innovative Medicine, Ajou University Medical Center, Suwon, Republic of Korea; 4 Department of Psychiatry and Behavioral Science, Seoul National University College of Medicine, Seoul, Republic of Korea; 5 Department of Research Planning, National Center for Mental Health, Seoul, Republic of Korea; 6 Department of Psychiatry, Seoul St. Mary’s Hospital, College of Medicine, The Catholic University of Korea, Seoul, Republic of Korea

**Keywords:** Neuroscience, Psychology

## Abstract

Although sleep disturbances are highly prevalent in adolescents, neuroimaging evidence on the effects of sleep disturbances on their developing brains remains limited. Therefore, we explored gray matter volumes (GMVs) at the whole-brain level and investigated their relationship to sleep disturbances in a sample of Korean adolescents in the general population. We recruited participants from one middle school and high school. All participants and their legal guardians gave informed consent before participating in our study. We used component 5 of the Pittsburgh Sleep Quality Index to measure sleep disturbances and conducted a voxel-based morphometry-DARTEL procedure to measure GMVs. We performed partial correlation analyses to examine whether the GMVs were associated with sleep disturbances. A total of 56 adolescents participated in this study. Our results revealed that GMVs in multiple global regions were negatively correlated with sleep disturbances. Moreover, most of these identified regions belong to large-scale brain networks categorized by functional neuroimaging studies. We found an association between regional GMVs in multiple global regions involved in large-scale networks and the severity of sleep disturbances in the adolescent population. Based on this evidence and previous neuroimaging evidence, we suggest that structural alterations in the networks may be linked to sleep disturbances.

## Introduction

Sleep disturbances and their harmful effects have been increasingly reported in many children and adolescents worldwide^1^. In a representative sample of 17,102 children and adolescents, approximately 20% reported sleep disturbances and an additional 13% reported difficulties falling asleep^1^. Similarly, 38% of 1,000 school going adolescents suffered from sleep disturbances^2^. A recent population-based study reported that 26% of 1,180 Brazilian children and adolescents aged 0–19 years experienced sleep disturbances, had inadequate sleep habits, and had sleep durations lower than the recommended duration^3^.

While sleep disturbances in adolescents may seem normative, sleep problems are known to be associated with a wide spectrum of physical and mental health problems, such as obesity, growth hormone deficiency, substance abuse, depression, suicidality, anxiety, difficulties with impulse control, and the compulsive use of social media and video games^4–12^. Moreover, strong associations between sleep problems and various cognitive functions, including memory, learning, and attention, have been reported by an established body of previous research^13–15^. Temporary sleep deprivation has also been shown to have a negative impact on sustained attention, working memory and executive function in healthy adolescents^14^.

Adolescence is a critical stage for normative brain development, as significant changes to brain structure, function and connectivity occur during this stage^16^. A large body of neuroimaging studies have investigated a wide variety of brain and neurocognitive development conditions and have demonstrated age-related effects and sex differences in gray matter volume (GMV) during adolescence^17–20^. Similarly, dramatic changes to sleep patterns (e.g., sleep duration, sleep shifts toward evening hours) take place during adolescence^8,9^. Inadequate sleep patterns, such as late bedtimes, short sleep durations, and poor sleep quality, have been frequently reported by adolescents in contemporary societies^15,21,22^. Delayed bedtimes and early morning awakenings for school substantially contribute to an accumulation of sleep debt during the school week^23^. Moreover, such sleep patterns are known to have detrimental effects on adolescents’ academic success and health, according to many epidemiological and experimental studies on sleep restriction^4,23^. Poor school performance and its correlations with both short and late sleep hours have been demonstrated by previous epidemiological studies with representative samples of adolescents^23–25^. The authors of these studies have postulated that such correlations might possibly be explained by cognitive processes, including attention and executive functions, which appear to be sensitive to insufficient sleep.

Although sleep disturbances have been identified as a serious public health concern, evidence of the effects of sleep disturbances on the developing brains of adolescents remains limited and unclear. In recent years, a neuroimaging approach to this topic has gained popularity, since it has the potential to reveal underlying neurological mechanisms of sleep problems, including sleep disturbances. A recent systematic review investigated the influences of sleep on the development of brain functions and structures in healthy children and adolescents (1–17 years of age)^15^. In this review, inadequate sleep was strongly associated with neuroanatomical structures and functions of the developing adolescent brain. Similarly, structural alterations in multiple brain regions have been found in adults with sleep problems, mainly those with primary insomnia^26–30^. These alterations have appeared mainly in the hippocampus, orbitofrontal cortex, and frontal and middle temporary gyri^31^. Additionally, a growing body of functional neuroimaging studies have demonstrated the relationship between aberrant regional brain activities and sleep problems, with sex differences across the whole brain^29,32^.

Although more attention should be given to children and adolescents, who have developing brains, most neuroimaging studies on sleep problems have focused on adults or patients with sleep problems^33^. However, it is known that general demographic factors (e.g., age, sex) profoundly influence the structure of the brain^34^. As brain development occurs profoundly during adolescence, it is important to explore how sleep disturbances adversely impact brain maturation, considering the high prevalence of sleep disturbances in this population^15^.

Additionally, convergent non-neuroimaging evidence suggests that sleep disturbances are linked to many symptoms (e.g., poor cognitive performance), which suggests that there may be a wide range of regions affected by sleep disturbances^35,36^. Nevertheless, in many previous neuroimaging studies, only specific regions of interest (e.g., the orbitofrontal cortex) were explored. Thus, potential structural alterations at the whole-brain network level may be relatively less explored, especially in the general adolescent population. Although the region of interest (ROI) method can provide important findings and is preferred for many neuroimaging studies, this method does not elucidate whether sleep disturbances can or do potentially affect other regions throughout the whole brain. An investigation of sleep disturbances and their relationship with other regions at the whole-brain level may be more appropriate for clarifying the effects of sleep disturbances on other global regions since multiple comparisons and corrections (e.g., type 1 error correction) can be performed with this method, allowing us to detect more statistically significant results. This neuroimaging approach may also help us gain a better comprehensive understanding of the health implications of sleep disturbances during adolescence. Determining the structural manifestations of sleep disturbances on the developing brain structure in adolescents may be crucial for developing more appropriate interventions specifically designed for this population as well as increasing public awareness of the importance of healthy sleep during adolescence.

### The present study

Considering the above concerns, we used the voxel-based morphometry (VBM)-DARTEL procedure to explore and assess the whole brain to investigate associations between regional gray matter volumes (rGMVs) and sleep disturbances in a sample of Korean adolescents from the general population.

## Results

### Demographic and clinical characteristics of the participants

Sixty-four adolescents aged between 12 and 17 volunteered to participate in this study. Of the 64 participants, 8 participants were excluded from the statistical analyses due to their incomplete responses, so a total of 56 participants were included in the analyses (32 males;24 females). The mean age ± standard deviation (SD) of the total sample was 14.71±1.37 years. The proportion of male participants (57.1%) was slightly larger than that of female participants (42.9%). A sex difference was observed in age (*p* = 0.02), as shown in Table 1. The total study population had an average score of 14.5 on component 5 of the Pittsburgh Sleep Quality Index (PSQI). As illustrated in Table 2, compared to female adolescents, male adolescents had large GMVs in all the identified regions, except the left inferior parietal lobule and the right postcentral gyrus. The total intracranial volume (TIV) was also relatively high in the male adolescents.

**Table 1:** Demographic and clinical characteristics of the study population.

Characteristics	Total	Sex		*t*	*p*^a^
		Male	Female		
Age (mean, *SD*^b^)	14.71 (1.37)	15.06 (1.50)	14.25 (1.03)	−2.40	0.020
Sleep disturbances^c^ (mean, *SD*)	14.45 (5.73)	14.63 (5.93)	14.21 (5.58)	−0.27	0.791

Total N = 56; Male N = 32 (57.14%); Female N = 24 (42.86%).

^a^2-tailed.

^b^Standard deviation.

^c^The total score of Component 5 of the Pittsburgh Sleep Quality Index Questionnaire; score range: 0–27.

**Table 2:** Sex differences in the gray matter volume of the brain regions.

Brain regions^a^	Sex		*p*
	Male	Female	
Rt. opercular inferior frontal gyrus	0.44 (0.05)	0.41 (0.04)	0.017
Rt. triangular inferior frontal gyrus	0.33 (0.04)	0.31 (0.03)	0.021
Rt. medial superior frontal gyrus	0.41 (0.05)	0.38 (0.04)	0.003
Lt. inferior parietal lobule	0.50 (0.06)	0.47 (0.06)	0.072
Lt. angular gyrus	0.50 (0.06)	0.44 (0.05)	0.001
Rt. inferior orbitofrontal gyrus	0.41 (0.04)	0.380 (0.04)	0.003
Rt. middle frontal gyrus	0.44 (0.05)	0.39 (0.04)	0.004
Lt. middle orbitofrontal cortex	0.50 (0.07)	0.45 (0.05)	0.004
Rt. middle orbitofrontal cortex	0.45 (0.06)	0.40 (0.04)	0.002
Rt. superior occipital gyrus	0.38 (0.05)	0.35 (0.04)	0.025
Rt. postcentral gyrus	0.35 (0.04)	0.33 (0.03)	0.068
Rt. insula	0.57 (0.05)	0.53 (0.04)	0.001
Total intracranial volume	1.47 (0.11)	1.35 (0.10)	<0.001

Abbreviations: Rt. = right; Lt. = left; SD = Standard Deviation.

^a^mean (SD).

### Correlation analysis

**Figure 1:**
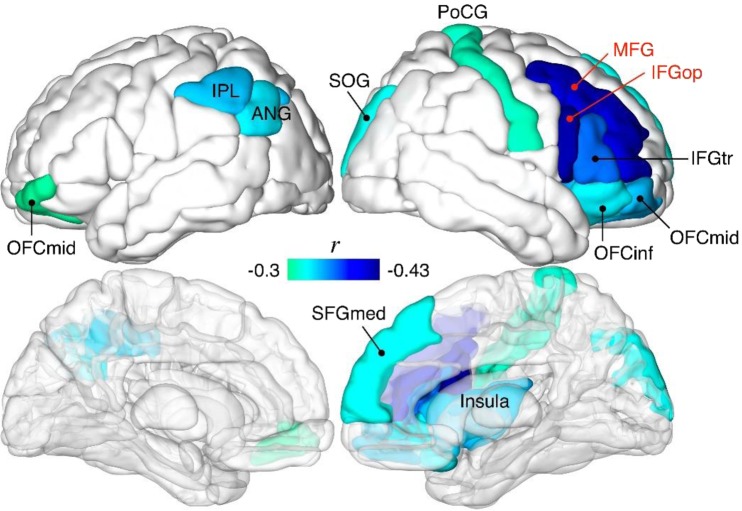
Association between rGMV and sleep disturbances (partial correlation; covariates = age, sex, TIV). All colored regions are significant with an FDR of <0.2 (red texts: FDR of <0.08). Abbreviations: OFCmid, middle orbitofrontal cortex; OFCinf, inferior orbitofrontal gyrus; IFGop, opercular inferior frontal gyrus; IFGtr, triangular inferior frontal gyrus; MFG, middle frontal gyrus; SFGmed, medial superior frontal gyrus; PoCG, postcentral gyrus; SOG, superior occipital gyrus; IPL, inferior parietal lobule; ANG, angular gyrus.

Regional gray matter volumes (rGMV) showed significant negative correlations with the severity of sleep disturbances at multiple regions in the frontal, occipital, postcentral, and parietal lobes and insula, even after the adjustment for covariates (i.e., age, sex, TIV). The regions were largely lateralized to the right hemisphere, except for the inferior parietal, angular, and middle orbitofrontal gyri. We described all partial correlation values and the significance values, including both uncorrected and FDR-corrected p-values, in Table 3. Figure 1 shows the correlations between the rGMVs and severity of sleep disturbances in our study population.

**Table 3:** Results of the partial correlation analyses between sleep disturbances and gray matter volumes (covariates = age, sex, TIV).

Brain regions	Network	Sleep disturbances		
		Pearson’s *r*	Uncorrected *p*	FDR corrected *p*
Rt. opercular inferior frontal gyrus	DMN	−0.43	0.001	0.078
Rt. triangular inferior frontal gyrus	DMN	−0.37	0.006	0.153
Rt. medial superior frontal gyrus	DMN	−0.33	0.017	0.153
Lt. inferior parietal lobule	DMN	−0.35	0.010	0.153
Lt. angular gyrus	CEN	−0.34	0.013	0.153
Rt. inferior orbitofrontal gyrus	CEN	−0.34	0.014	0.153
Rt. middle frontal gyrus	CEN	−0.42	0.002	0.078
Lt. middle orbitofrontal cortex	SN	−0.31	0.026	0.194
Rt. middle orbitofrontal cortex	SN	−0.35	0.010	0.153
Rt. insula	SN	−0.35	0.010	0.153
Rt. superior occipital gyrus	VN	−0.33	0.017	0.153
Rt. postcentral gyrus	SSN	−0.31	0.022	0.179

Abbreviations: Rt. = right; Lt. = left; DMN = default mode network; CEN = central executive network; SN = salience network; VN = visual network; SSN = somatosensory network.

## Discussion

In the present study, the assessment was not solely based on a single region of interest; rather, it focused on the whole brain. Using VBM-DARTEL, we found an association between the rGMVs in global brain regions and the severity of sleep disturbances in a sample of Korean adolescents in the general population. Its magnitude remained strong, even after we controlled for relevant covariates (i.e., age, sex, TIV) and type I errors. Interestingly, we also found that these identified global regions are components of large-scale brain networks categorized by a large body of functional magnetic resonance imaging (fMRI) studies with adult study populations in clinical settings and nonclinical settings^32,35–43^. The networks include the default mode network (DMN), central executive network (CEN), visual network (VN), somatosensory network (SSN), and salience network (SN). In recent years, altered functional connectivity in these networks has increasingly been revealed by many neuroimaging studies on different domains of sleep problems^44–46^. Comparably, the relationships between the structural alterations in some core regions of these networks and different domains of sleep problems have been increasingly reported by magnetic resonance imaging (MRI) studies conducted in adult populations^15,26–28^. Based on all of this evidence, we discuss in this section altered GMVs and their relationship with sleep disturbances from this large-scale brain network perspective.

Table 3 lists the multiple brain regions that are involved in the DMN. In our study, adolescents with higher levels of sleep disturbances had decreased volumes in the right opercular inferior frontal gyrus, the right triangular inferior frontal gyrus, the right medial superior frontal gyrus, and the left inferior parietal lobule. Among these regions, the right inferior frontal gyrus had the strongest association with sleep disturbances. The inferior parietal lobule is regarded as one of the core regions in this network^47^. Several functional and structural neuroimaging studies and an electroencephalogram (EEG) study conducted in adolescent populations have also revealed associations between sleep problems, including insufficient sleep and poor sleep quality, altered GMVs and atypical activation in multiple brain regions that are implicated in the DMN^16,23,48–51^. Similarly, a smaller GMV in the right middle superior frontal gyrus has also been shown to be correlated with a later bedtime on weekends in 14-year-old community adolescents^23^. The DMN is conventionally regarded as a network that is active when the brain is at rest, yet there is compelling evidence that the DMN also plays an important role in sleep as well as in other activities^37,52^. A growing body of structural and functional neuroimaging studies have demonstrated that altered GMVs and functional connectivity within the DMN are linked not only to sleep problems, but also to other symptoms of sleep problems, including poor cognitive functions^30,45,53^. Specifically, the prefrontal cortex is active not only during the deactivation of rapid eye movement (REM) sleep and the transition between wake and non-REM sleep but also in social cognitive processes such as decision making and self-regulation^37^.

As shown in Table 3, the right middle frontal gyrus, the angular gyrus, and the right inferior orbitofrontal gyrus are implicated with the CEN, which is responsible for decision making, working memory, judgment, and goal-oriented planning^35,37^. The orbitofrontal cortex is known to be one of the key nodes of this network^53^. Although we described here the angular gyrus as a region in the CEN, this region is also regarded as a hub for other networks (i.e., DMN, attention network) involved in various functions (e.g., self-processing, attention, semantic information processing)0^54^. Our findings corroborate those of a structural neuroimaging study that first demonstrated robust evidence on how sleep habits are associated with rGMVs and average academic performance during early adolescence^23^. A shorter time in bed during the weekdays has been shown to be strongly associated with a smaller GMV in the middle frontal gyrus^23^. Our findings also align with the results of a previous study that revealed smaller GMVs in the left orbitofrontal and parietal cortices in a sample of patients with chronic insomnia^26^.

Our findings also showed that the right superior occipital gyrus, which belongs to the VN, which is responsible for visual information processing, is also associated with sleep disturbances^36^. The left middle occipital gyrus and left lingual gyrus are known to be key nodes of the VN^44,55^. We found that the GMV of the right superior occipital gyrus was negatively correlated with the severity of sleep disturbances. A recent fMRI study reported altered emotion-dependent functional connectivity between the occipital cortex and other brain regions and its positive association with sleep duration in a sample of school-aged children^56^. In contrast, a recent neuroimaging study conducted in a sample of primary insomnia patients found an increased volume and altered functional connectivity in the left superior occipital gyrus^40^. Earlier fMRI studies demonstrated hyperarousal activation in the bilateral occipital gyrus during sleep deprivation and in individuals with primary insomnia^37,44,55,57^. This discrepancy in previous results might be due to the heterogeneity of the study populations as well as the scarcity of available data. Therefore, these results should be corroborated by additional investigations with larger samples sizes of adolescents in the general population.

Similarly, we found decreased volume in the right postcentral gyrus, a main receptive region for external stimuli, which is located in the primary somatosensory cortex^28^. The SSN plays a critical role in processing somatosensory input and integrating sensory and motor signals for skilled movement^58^. Consistent with our findings, an earlier structural MRI study demonstrated a significant reduction in gray matter in the bilateral postcentral gyrus in patients with primary insomnia^28^. Specifically, this result was related to prolonged sleep latency as well as difficulty in sleep initiation. This study suggested that the association may be explained by a decreased capacity to disengage from external information processing^28^.

In our study, decreased GMVs in the bilateral middle orbitofrontal cortex and the right insula also had strong associations with more severe sleep disturbances. These regions are considered key nodes of the SN, a network that plays a major role in detecting, filtering, and processing external and internal stimuli as well as in attentional filtering and orienting and emotional processing^42,43^. Our findings support the existing robust evidence that this region is closely linked to sleep disturbances^26,28^. The first VBM study^26^ that compared the rGMVs of 24 adults with chronic primary insomnia with those of control subjects without sleep problems and showed a strong negative correlation between GMV in the left orbitofrontal cortex and subjective severity of insomnia. Furthermore, our findings pertaining to the insula support established neuroimaging evidence on the strong link between this region and different domains of sleep problems (e.g., poor sleep quality)^37,59–62^. A recent study^63^ found a significant correlation between sleep quality and depression/anxiety among 370 college students. Moreover, the mediating role of the GMV in the right insula in this correlation was revealed, which raises the possibility that depression/anxiety may affect sleep quality through volumetric variation in the right insula. Collectively, fMRI studies have provided compelling evidence of altered functional connectivity in this network in individuals with sleep problems^39,43^. Furthermore, the SN appears to play a crucial role in alternately activating the CEN and the DMN^39^. A recent systematic review on obstructive sleep apnea (OSA) and insomnia revealed altered functional connectivity in the SN as well as its link to the DMN and the CEN, and it highlighted the functional integration of the large-scale brain networks^39^.

In the current study, by assessing the whole brain in a sample of adolescents in the general population, we found that structural alterations in multiple regions of the large-scale brain networks categorized by fMRI studies were associated with sleep disturbances. From a neuroimaging perspective, it has been postulated that these networks interact with one another simultaneously^64,65^. Conversely, disruptions in these networks may contribute to a wide range of problems^65^. An established body of both functional and structural neuroimaging research has revealed altered functional connectivity and GMVs of the networks in adults and patients with diverse sleep problems^37,40,42,66^. Liu *et al.*^37^ made the first attempt to characterize the topological architecture of whole-brain functional networks in individuals with primary insomnia, using a seed-based functional connectivity approach. The authors revealed a pattern of functional deficits in the DMN, CEN, and SN.

Furthermore, the strength of the association between rGMVs in our identified regions of the networks and levels of sleep disturbances remained strong, even after we adjusted for age, sex, and TIV. This result is noteworthy since our findings suggest that rGMVs in broad regions throughout the brain might be affected by sleep disturbances prior to adulthood. This result is consistent with that in a recent systematic review on the effects of sleep on developing brain functions and structures in the pediatric and adolescent population^15^. In this review, multiple epidemiological studies have reported an association between sleep and gray and/or white matter volumes in school-aged children and adolescents. For instance, smaller GMVs were associated with later bedtimes on the weekends in adolescents^23^. Likewise, high levels of subjectively measured sleep variability and its association with low white matter integrity were observed during mid- to late- adolescence, indicating that high sleep variability may impair white matter development^67^.

Based on these results and previous structural and functional neuroimaging evidence, we suggest that GMVs in multiple regions of the large-scale networks may already be affected by sleep disturbances prior to early adulthood. Moreover, it is possible that the relationship between structural alterations in the large-scale networks and sleep disturbances may be involved in a wide variety of symptoms (e.g., poor cognitive performance), as these networks are known to be responsible for a broad range of functions. Considering the limited neuroimaging evidence on this relationship in adolescents, the results of our study may provide new insights into determining the structural manifestations of sleep disturbances in the developing brains of adolescents in the general population as well as developing a comprehensive understanding of sleep disturbances and their relationships with the brain structures of adolescents. In accordance with our findings, the seriousness of sleep problems and the importance of healthy sleep should be encouraged and prioritized in the adolescent population. Health care providers and clinicians should emphasize healthy sleep habits and sufficient time for sleep, as sleep may be essential for developing brain structures and functions during adolescence.

Despite the novelty of the current study, our findings have some limitations that should be taken into account. First, we measured the severity of sleep disturbances with a single component of the PSQI, which was a major limitation of our study and may limit the generalizability of our findings. Although this component has been validated and has been shown to have high specificity and sensitivity, a previous study showed a nonsignificant correlation between the PSQI components and actigraphy results in a nonclinical sample^68^. Second, our findings may not be generalizable to other adolescent populations because sampling bias may be present due to the method of participant recruitment that we used (i.e., participants were recruited from one middle school and one high school).

Third, there may be other potential confounders (e.g., depression, anxiety) that might have contributed to the magnitude of the association between the rGMVs and sleep disturbances^69^. Last, our findings were exploratory and we did not present any specific hypotheses, so our findings may have limited contributions toward gaining a better understanding of the association of GMVs with sleep disturbances. We therefore recommend that future studies with VBM group analyses between adolescents with sleep disturbances and those without sleep disturbances and correlation analyses are conducted to elucidate the relationships between structural alterations in the large-scale networks and sleep disturbances. A seed-based structural covariance analysis may also be helpful to examine these relationships in adolescent populations. In addition to this analysis method, we recommend that additional studies are conducted to examine whether GMVs in our identified regions become relatively smaller with age in a sample of adolescents with sleep problems versus those without sleep problems.

To our knowledge, at present, there is not enough data of the relationship between sleep disturbances and structural alterations in brain regions in adolescent populations. Therefore, we made an attempt to investigate whether there is an association between rGMVs and the severity of sleep disturbances in a sample of Korean adolescents from the general population. Our results revealed that rGMVs in multiple global regions were negatively correlated with the severity of sleep disturbances. The magnitude of this association remained strong, even after controlling for multiple covariates. Combined with previous structural and functional neuroimaging evidence, we suggest that the structural changes to gray matter in multiple regions that are involved in the large-scale networks may be linked to sleep disturbances. Moreover, we recommend that an investigation of both functional and structural alterations in the regions of the major networks may be beneficial to tackle underlying neurological mechanisms of sleep disturbances.

## Methods

### Study population

We recruited adolescents aged from 12 to 18 years from one middle school and one high school in Seoul, South Korea. After the school principals approved our study, we visited the students and teachers at the schools to explain the study’s objectives and guaranteed confidentiality of their information. We also mailed letters to the parents of potential participants containing brief information about the study along with the contact information of the principal investigator. The letter also stated that they would be informed of the results of our study following the completion of the analyses. All participants and their parents or legal guardians provided informed consent before taking part in the study.

All participants who participated in our study (a) were capable of fully understanding the description of and following the instructions of the present study; (b) had no possibility of pregnancy prior to the study; (c) did not consume drugs that could significantly affect their sleep and waking conditions; and (d) showed no expected problems in brain imaging and psychological tests. Participants with any clear history of an acquired brain injury such as cerebral palsy, neurological disorders such as convulsive disorder, psychiatric disorders (including schizophrenia, bipolar disorder, or pediatric psychosis), developmental disorders (including autism or intellectual disabilities), learning disabilities, language impairments, or uncorrected sensory impairment were excluded from the analyses in our study. This study was approved by The Institutional Review Board for Human Subjects at Seoul National University Hospital and conducted in accordance with the Declaration of Helsinki.

### Study variables

#### Pittsburgh Sleep Quality Index (PSQI)

The PSQI is a self-rated questionnaire that assesses sleep quality and sleep disturbances over the past month^70^. It has been well validated and is widely used in both clinical and nonclinical settings^71,72^. It has demonstrated high levels of internal consistency and construct validity, with a Cronbach’s alpha of 0.8^73^. The 19 items provide a global sleep quality index score based on seven component scores: sleep quality, sleep latency, sleep duration, habitual sleep efficiency, sleep disturbances, the use of sleep medications, and daytime dysfunction. With each component equally weighted on a 0–3 scale, the range of the global score is from 0–21. Higher scores indicate worse global sleep quality.

For the present study, we used a single component, component 5, since we focused on sleep disturbances; component 5 measures the severity of sleep disturbances. Regarding the other components, we concluded that these components did not accurately reflect the sleep quality of Korean adolescents, considering there is a difference between their weekday sleep and weekend sleep due to a variety of external factors (e.g., early school start times, extra classes or private lessons at night) that could profoundly restrict their sleep duration^6,74^. Moreover, as the participants in our study did not take sleeping medications, component 6 was not used in our study. A recent study validated the single-factor scoring structure and psychometric properties of the PSQI in a sample of community-based adolescents^75^. Its finding of a single-factor model was consistent with other models used in previous studies conducted in adults and adolescents. The Cronbach’s alpha of each PSQI component was higher than 0.6 (α = 0.71 for component 5).

Component 5 consists of a set of 10 items that ask respondents how often they had trouble sleeping^70^. For each statement (e.g., I cannot get to sleep within 30 minutes, I wake up in the middle of the night or early morning), the response options were ‘not during the past month,’ ‘less than once a week,’ ‘once or twice a week,’ and ‘three or more times a week.’ The scores of sleep disturbance ranged from 0–27. Higher scores indicate more severe sleep disturbances.

#### Voxel-Based Morphometry (VBM) Analysis

To prevent circularity problems during the process of registration (i.e., tissue classification, spatial normalization, spatial smoothing), a refined VBM method has been introduced and implemented^31,76,77^. This method includes a new registration method called diffeomorphic anatomical registration involving exponentiated Lie algebra (DARTEL)^31^. This method provides clearer segmentation and better registration with regards to boundaries between gray matter and white matter compared to optimized VBM^78,79^. With this method, we conducted a VBM analysis using the SPM12 VBM-DARTEL procedure (SPM12, http://www.fil.ion.ucl.ac.uk/spm/,Wellcome Trust Centre for Neuroimaging, London, UK)^80^.

No abnormalities from motion or other artifacts were found in the T1-weighted images, which were inspected by a well-trained physician. The procedure for preprocessing the T1-weighted images included (i) manual reorientation to the anterior commissure, (ii) gray matter segmentation based on a standard tissue probability map provided from SPM, (iii) the creation of a study-specific template, spatial normalization with DARTEL to normalize individual images to the DARTEL template, modulation to adjust for volume signal changes during spatial normalization and (iv) spatial smoothing of the gray matter partitions with a Gaussian kernel of 8 mm full-width at half maximum. After preprocessing, the rGMV for each area was extracted by averaging the values in 116 brain regions from the AAL atlas^81^.

For this particular study, we used the standard adult SPM template instead of an age-specific template. While this application remains controversial^82^, several neuroimaging studies on developing brains have not only shown neuroanatomical differences between adults but also demonstrated valid neuroimaging results for children using the adult template^71,83–85^. Moreover, the standard adult SPM template enables us to compare or combine the results of previous studies conducted in adults or across different age groups^72,86,87^.

### Statistical analysis

All statistical analyses were performed using MATLAB-based custom software (MathWorks, Sherborn, MA, USA) and SPSS 20.0 for Windows (SPSS Inc., Chicago, IL, USA). Partial correlation analyses were carried out to assess which relevant covariates established by the previous literature were associated with sleep disturbances. The factors include the participant’s age, sex, TIV, and caffeine consumption^15,17,74^. As a next step, we performed partial correlation analyses (covariates; age, sex, and TIV) to investigate associations of rGMVs with the severity of sleep disturbances. A false discovery rate (FDR) threshold of <0.2 was determined to be significant for addressing multiple comparison issues^78^. FDR thresholding controls the expected proportion of false positives only among brain regions showing significance^78^. Although conservative levels of FDR (e.g., 0.01–0.05) can be used in neuroimaging studies, FDR control levels in the range of 0.1–0.2 are originally and practically known to be acceptable and have been applied in several neuroimaging studies^33,78,79,88,89^. FDR corrected p-values were calculated by using spm_P_FDR.m with all regional p-value inputs, which is a MATLAB code included in the SPM toolbox.

## References

[1] Schlarb AA, Gulewitsch MD, Weltzer V, Ellert U, Enck P (2015). Sleep Duration and Sleep Problems in a Representative Sample of German Children and Adolescents. Health (Irvine. Calif)..

[2] Saxena S, Koreti S, Gaur A (2016). Prevalence and Predictors of Sleep Wake Disturbances Among Adolescents. Int. J. Contemp. Med. Res..

[3] de Almeida GMF, Nunes ML (2019). Sleep characteristics in Brazilian children and adolescents: a population-based study. Sleep Med. X.

[4] Owens J (2014). Insufficient Sleep in Adolescents and Young Adults: An Update on Causes and Consequences. Pediatrics.

[5] Gomes GC (2017). Sleep quality and its association with psychological symptoms in adolescent athletes. Rev. Paul. Pediatr..

[6] Chiu HY, Lee HC, Chen PY, Lai YF, Tu YK (2018). Associations between sleep duration and suicidality in adolescents: A systematic review and dose–response meta-analysis. Sleep Med. Rev..

[7] Verkooijen S (2018). Sleep Disturbances, Psychosocial Difficulties, and Health Risk Behavior in 16,781 Dutch Adolescents. Acad. Pediatr..

[8] Conklin AI, Yao CA, Richardson CG (2019). Chronic sleep disturbance, not chronic sleep deprivation, is associated with self-rated health in adolescents. Prev. Med. (Baltim)..

[9] Raudsepp L, Vink K (2019). Brief report: Longitudinal associations between physical activity, sleep disturbance and depressive symptoms in adolescent girls. J. Adolesc..

[10] Bauducco SV, Salihovic S, Boersma K (2019). Bidirectional associations between adolescents’ sleep problems and impulsive behavior over time. Sleep Med. X.

[11] Kwon M, Park E, Dickerson SS (2019). Adolescent substance use and its association to sleep disturbances: a systematic review. Sleep Heal..

[12] Lima RA (2020). The synergic relationship between social anxiety, depressive symptoms, poor sleep quality and body fatness in adolescents. J. Affect. Disord..

[13] Kuula L (2015). Poor sleep and neurocognitive function in early adolescence. Sleep Med..

[14] Lo JC, Ong JL, Leong RLF, Gooley JJ, Chee MWL (2016). Cognitive Performance, Sleepiness, and Mood in Partially Sleep Deprived Adolescents: The Need for Sleep Study. Sleep.

[15] Dutil C (2018). Influence of sleep on developing brain functions and structures in children and adolescents: A systematic review. Sleep Med. Rev..

[16] Goldstone A (2018). The mediating role of cortical thickness and gray matter volume on sleep slow-wave activity during adolescence. Brain Struct. Funct..

[17] Gennatas ED (2017). Age-related effects and sex differences in gray matter density, volume, mass, and cortical thickness from childhood to young adulthood. J. Neurosci..

[18] Group BDC (2012). Total and regional brain volumes in a population-based normative sample from 4 to 18 years: the NIH MRI Study of Normal Brain Development. Cereb. Cortex.

[19] Gogtay N (2004). Dynamic mapping of human cortical development during childhood through early adulthood. Proc. Natl. Acad. Sci..

[20] Shaw, P. *et al*. Neurodevelopmental trajectories of the human cerebral cortex. *J. Neurosci*. **28**, (2008). 10.1523/JNEUROSCI.5309-07.2008 PMC6671079 18385317

[21] Matricciani L (2017). Past, present, and future: trends in sleep duration and implications for public health. Sleep Heal..

[22] Bin, Y. S., Marshall, N. S. & Glozier, N. Secular trends in adult sleep duration:a systematic review. *Sleep Med. Rev*. **16**, (2012). 10.1016/j.smrv.2011.07.003 22075214

[23] Urrila AS (2017). Sleep habits, academic performance, and the adolescent brain structure. Sci. Rep..

[24] Merikanto I, Lahti T, Puusniekka R, Partonen T (2013). Late bedtimes weaken school performance and predispose adolescents to health hazards. Sleep Med..

[25] Hysing, M., Pallesen, S. & Stormark, K. M. Adolescents and insomnia Sleep patterns and insomnia among adolescents: a population-based study. 549–556, 10.1111/jsr.12055 (2013). 10.1111/jsr.12055 23611716

[26] Altena E, Vrenken H, Van Der Werf YD, Van Den Heuvel OA, Van Someren EJW (2010). Reduced Orbitofrontal and Parietal Gray Matter in Chronic Insomnia: A Voxel-Based Morphometric Study. Biol. Psychiatry.

[27] Heidbreder A (2017). Gray matter abnormalities of the dorsal posterior cingulate in sleep walking. Sleep Med..

[28] Joo EY (2013). Brain Gray Matter Deficits in Patients with Chronic Primary Insomnia. Sleep.

[29] Li Z (2018). Disrupted brain network topology in chronic insomnia disorder: A resting-state fMRI study. NeuroImage Clin..

[30] Shi Y (2017). A Meta-analysis of Voxel-based Brain Morphometry Studies in Obstructive Sleep Apnea. Sci. Rep..

[31] Li M (2018). Altered gray matter volume in primary insomnia patients: a DARTEL-VBM study. Brain Imaging Behav..

[32] Wu Y (2018). Abnormal topology of the structural connectome in the limbic cortico-basal-ganglia circuit and default-mode network among primary insomnia patients. Front. Neurosci..

[33] Jung KI (2019). Cerebellar Gray Matter Volume, Executive Function, and Insomnia: Gender Differences in Adolescents. Scentific Reports.

[34] Kurth F, Luders E, Angeles L (2015). Voxel-Based Morphometry. Brain MappingAn Encycl. Ref..

[35] Kang L (2016). Altered resting-state functional organization within the central executive network in obsessive-compulsive disorder. Psychiatry Clin. Neurosci..

[36] Yang YL, Deng HX, Xing GY, Xia XL, Li HF (2015). Brain functional network connectivity based on a visual task: Visual information processing-related brain regions are significantly activated in the task state. Neural Regen. Res..

[37] Liu X, Zheng J, Liu BX, Dai XJ (2018). Altered connection properties of important network hubs may be neural risk factors for individuals with primary insomnia. Sci. Rep..

[38] Tashjian SM, Goldenberg D, Monti MM, Galván A (2018). Sleep quality and adolescent default mode network connectivity. Soc. Cogn. Affect. Neurosci..

[39] Hua K (2018). Aberrant Effective Connectivity of the Right Anterior Insula in Primary Insomnia. Front. Neurol..

[40] Li, G. *et al*. Magnetic resonance study on the brain structure and resting-state brain functional connectivity in primary insomnia patients. *Med. (United States)* **97**, (2018). 10.1097/MD.0000000000011944 PMC6113012 30142814

[41] Liu CH (2018). Increased salience network activity in patients with insomnia complaints in major depressive disorder. Front. Psychiatry.

[42] Lei Y (2015). Large-Scale Brain Network Coupling Predicts Total Sleep Deprivation Effects on Cognitive Capacity. PLoS One.

[43] Khazaie H (2017). Functional reorganization in obstructive sleep apnoea and insomnia: A systematic review of the resting-state fMRI. Neurosci. Biobehav. Rev..

[44] Liu X (2016). Gender Differences in Regional Brain Activity in Patients with Chronic Primary Insomnia: Evidence from a Resting-State fMRI Study. J. Clin. Sleep Med..

[45] Santarnecchi, E. *et al*. Age of insomnia onset correlates with a reversal of default mode network and supplementary motor cortex connectivity. *Neural Plast*. **2018**, (2018). 10.1155/2018/3678534 PMC5901935 29808082

[46] Dong X (2018). Rest but busy: Aberrant resting-state functional connectivity of triple network model in insomnia. Brain Behav..

[47] Buckner RL, Andrews-Hanna JR, Schacter DL (2008). The brain’s default network: Anatomy, function, and relevance to disease. Ann. N. Y. Acad. Sci..

[48] Beebe DW, DiFrancesco MW, Tlustos SJ, McNally KA, Holland SK (2009). Preliminary fMRI findings in experimentally sleep-restricted adolescents engaged in a working memory task. Behav. Brain Funct..

[49] Robinson JL, Erath SA, Kana RK, El-Sheikh M (2018). Neurophysiological differences in the adolescent brain following a single night of restricted sleep. Dev. Cogn. Neurosci..

[50] Buchmann, A. *et al*. EEG sleep slow-wave activity as a mirror of cortical maturation. *Cereb. Cortex* **21**, (2011). 10.1093/cercor/bhq129 20624840

[51] Taki Y (2012). Sleep duration during weekdays affects hippocampal gray matter volume in healthy children. Neuroimage.

[52] Andrews-Hanna, J R; Smallwood, Jonathan and Spreng, N. The default network and self-generated thought:component processes, dynamic control, and clinical relevance. **1316**, 29–52 (2014). 10.1111/nyas.12360 PMC4039623 24502540

[53] Chen L (2018). Topological Reorganization of the Default Mode Network in Severe Male Obstructive Sleep Apnea. Front. Neurol..

[54] Tanaka S, Kirino E (2019). Increased Functional Connectivity of the Angular Gyrus During Imagined Music Performance. Front. Hum. Neurosci..

[55] Gao L (2015). Frequency-dependent changes of local resting oscillations in sleep-deprived brain. PLoS One.

[56] Reidy BL, Hamann S, Inman C, Johnson KC, Brennan PA (2016). Decreased sleep duration is associated with increased fMRI responses to emotional faces in children. Neuropsychologia.

[57] Dai X-J (2012). Gender differences in brain regional homogeneity of healthy subjects after normal sleep and after sleep deprivation: A resting-state fMRI study. 2Sleep Med..

[58] Borich MR, Brodie SM, Gray WA, Ionta S, Boyd LA (2015). Understanding the role of the primary somatosensory cortex: Opportunities for rehabilitation. Neuropsychologia.

[59] Li X (2017). Increased interhemispheric resting-state functional connectivity in healthy participants with insomnia symptoms. Medicine (Baltimore)..

[60] Uy JP, Galván A (2017). Neuropsychologia Sleep duration moderates the association between insula activation and risky decisions under stress in adolescents and adults. Neuropsychologia.

[61] Liu C, Liu C, Zhang J, Yuan Z, Tang L (2016). Reduced spontaneous neuronal activity in the insular cortex and thalamus in healthy adults with insomnia symptoms. Brain Res..

[62] Liu C, Liu C, Zhu X, Fang J, Lu S (2018). Increased Posterior Insula-Sensorimotor Connectivity Is Associated with Cognitive Function in Healthy Participants with Sleep Complaints. Front. Hum. Neurosci..

[63] Yin, H., Zhang, L., Li, D., Xiao, L. & Cheng, M. The gray matter volume of the right insula mediates the relationship between symptoms of depression/anxiety and sleep quality among college students. *J. Health Psychol*. (2019). 10.1177/1359105319869977 31411064

[64] Friston KJ (2005). Models of Brain Function in Neuroimaging. Annu. Rev. Psychol..

[65] Smith DV, Gseir M, Speer ME, Delgado MR (2016). Toward a cumulative science of functional integration: A meta-analysis of psychophysiological interactions. Hum. Brain Mapp..

[66] Yu H-H (2018). Aberrant brain functional connectome in patients with obstructive sleep apnea. Neuropsychiatr. Dis. Treat..

[67] Telzer EH, Goldenberg D, Fuligni AJ, Lieberman MD, Gálvan A (2015). Developmental Cognitive Neuroscience Sleep variability in adolescence is associated with altered brain development. Accid. Anal. Prev..

[68] Grandner MA, Kripke DF, Yoon I-Y, Youngsted SD (2006). Criterion validity of the Pittsburgh Sleep Quality Index: Investigation in a non-clinical sample. Sleep Biol. Rhythms.

[69] Baddam S, Canapari C, van Noordt S, Crowley M (2018). Sleep Disturbances in Child and Adolescent Mental Health Disorders: A Review of the Variability of Objective Sleep. Markers. Med. Sci..

[70] Buysse DJ, Reynolds CF, Monk TH, Berman SR, Kupfer DJ (1988). The Pittsburgh Sleep Quality Index: A New Instrument for Psychiatric Practice and Research. Psychiatry Res..

[71] Poldrack RA (2010). Interpreting developmental changes in neuroimaging signals. Hum. Brain Mapp..

[72] Weiss Y, Booth JR (2017). Neural correlates of the lexicality effect in children. Brain Lang..

[73] Carpenter JS, Andrykowski MA (1998). Psychometric Evaluation of The Pittsburgh Sleep Quality Index. J. Psychosom. Res..

[74] Bartel KA, Gradisar M, Williamson P (2015). Protective and risk factors for adolescent sleep: A meta-analytic review. Sleep Med. Rev..

[75] Raniti MB, Waloszek JM, Schwartz O, Allen NB, Trinder J (2018). Factor structure and psychometric properties of the Pittsburgh Sleep Quality Index in community-based adolescents. Sleep Res. Soc..

[76] Ashburner J (2007). A fast diffeomorphic image registration algorithm. Neuroimage.

[77] Takao H (2010). Cerebral asymmetry in patients with schizophrenia: a voxel-based morphometry (VBM) and diffusion tensor imaging (DTI) study. J. Magn. Reson. Imaging.

[78] Genovese CR, Lazar NA, Nichols T (2002). Thresholding of statistical maps in functional neuroimaging using the false discovery rate. Neuroimage.

[79] Molteni E (2017). A diffusion tensor magnetic resonance imaging study of paediatric patients with severe non‐traumatic brain injury. Dev. Med. Child Neurol..

[80] Edelman GM, Tononi G, Sporns O, Reeke GN, Friston KJ (1994). Value-dependent selection in the brain: Simulation in a synthetic neural model. Neuroscience.

[81] Tzourio-Mazoyer N (2002). Automated anatomical labeling of activations in SPM using a macroscopic anatomical parcellation of the MNI MRI single-subject brain. Neuroimage.

[82] Richards JE, Sanchez C, Phillips-Meek M, Xie W (2016). A database of age-appropriate average MRI templates. Neuroimage.

[83] Burgund E (2002). The feasibility of a common stereotactic space for children and adults in fMRI studies of development. Neuroimage.

[84] Muzik O, Chugani DC, Juhasz C, Shen C, Chugani HT (2000). Statistical parametric mapping: Assessment of application in children. Neuroimage.

[85] Kang HC, Burgund ED, Lugar HM, Petersen SE, Schlaggar BL (2003). Comparison of functional activation foci in children and adults using a common stereotactic space. Neuroimage.

[86] Ross P, de Gelder B, Crabbe F, Grosbras MH (2019). Emotion modulation of the body-selective areas in the developing brain. Dev. Cogn. Neurosci..

[87] Verdejo-Román JL (2019). Maternal prepregnancy body mass index and offspring white matter microstructure: results from three birth cohorts. Int. J. Obes..

[88] Yu, Q. *et al*. Lipidome alterations in human prefrontal cortex during development, aging, and cognitive disorders. *Mol. Psychiatry* (2018). 10.1038/s41380-018-0200-8 PMC7577858 30089790

[89] Gordon BA (2018). Cross-sectional and longitudinal atrophy is preferentially associated with tau rather than amyloid β positron emission tomography pathology. Alzheimer’s Dement. Diagnosis, Assess. Dis. Monit..

